# Harnessing the capabilities of VCSELs: unlocking the potential for advanced integrated photonic devices and systems

**DOI:** 10.1038/s41377-024-01561-8

**Published:** 2024-09-03

**Authors:** Guanzhong Pan, Meng Xun, Xiaoli Zhou, Yun Sun, Yibo Dong, Dexin Wu

**Affiliations:** 1 Institute of Microelectronics, Chinese Academy of Sciences, Beijing, China; 2 Institute of Photonic Chips, University of Shanghai for Science and Technology, Shanghai, China

**Keywords:** Semiconductor lasers, Micro-optics, Optical manipulation and tweezers, Integrated optics

## Abstract

Vertical cavity surface emitting lasers (VCSELs) have emerged as a versatile and promising platform for developing advanced integrated photonic devices and systems due to their low power consumption, high modulation bandwidth, small footprint, excellent scalability, and compatibility with monolithic integration. By combining these unique capabilities of VCSELs with the functionalities offered by micro/nano optical structures (e.g. metasurfaces), it enables various versatile energy-efficient integrated photonic devices and systems with compact size, enhanced performance, and improved reliability and functionality. This review provides a comprehensive overview of the state-of-the-art versatile integrated photonic devices/systems based on VCSELs, including photonic neural networks, vortex beam emitters, holographic devices, beam deflectors, atomic sensors, and biosensors. By leveraging the capabilities of VCSELs, these integrated photonic devices/systems open up new opportunities in various fields, including artificial intelligence, large-capacity optical communication, imaging, biosensing, and so on. Through this comprehensive review, we aim to provide a detailed understanding of the pivotal role played by VCSELs in integrated photonics and highlight their significance in advancing the field towards efficient, compact, and versatile photonic solutions.

## Introduction

The field of integrated photonic devices has witnessed significant progress in recent years, largely driven by potential applications and by the increasing demand for compact, efficient, and multifunctional optoelectronic systems^1–4^. The ability to integrate multiple optical functionalities onto a single chip holds great promise for enhancing device performance, reducing power consumption, and unlocking new opportunities to revolutionize various areas ranging from artificial intelligence and optical communication to sensing and imaging systems.

Semiconductor lasers, including edge emitting lasers (EELs) and vertical cavity surface emitting lasers (VCSELs), have gained considerable attention in the context of integrated photonics due to their small size, light weight, and high power conversion efficiency. Compared with VCSELs, EELs can generally achieve higher output power for a single emitter, which is attractive for long-distance sensing like LiDAR. Besides, EELs are easier to achieve long wavelengths transparent for silicon materials, and their edge-emitting configuration makes them easier to integrate with in-plane horizontal waveguides, making them be usually used in two-dimensional (2D) in-plane integration like silicon photonics integrated circuits (PICs). Unlike EELs, VCSELs emit light perpendicular to the surface of the wafer, which are more suitable for three-dimensional (3D) vertically stacked integration. Nevertheless, methods such as using end-to-end coupling^5^, tilted configuration with grating couplers^6^, or 45^o^ microreflectors^7^ can be used for VCSELs to achieve the integration with in-plane horizontal waveguides in silicon photonics. More importantly, compared to EELs, VCSELs are of more symmetric beam profile, lower threshold, higher temperature stability, and easier of achieving 2D laser arrays^8,9^, which are getting more and more intention in integrated photonics. Besides, the wafer-normal emission makes VCSELs highly scalable and compatible with wafer-scale manufacturing processes, enabling scalable integrated photonic devices and mass production with relatively low cost. Moreover, VCSELs can achieve relatively high bandwidths and support high-speed direct modulation, making them suitable for integrated photonic devices/systems that require high-speed data transmission. These charming advantages make VCSELs very suitable candidates for integrated photonics, especially for vertically stacked photonics integration.

So far, VCSEL-based integrated photonics have been widely investigated. The monolithic vertically stacked integration capability of VCSELs allows for compact integration with other optical components, such as micro lenses^10–15^ and diffractive optical elements^16–18^, laying the foundation for complex and compact integrated photonic systems. Various fabrication techniques, such as lithography^17^ and etching^13,14^, have been explored to achieve on-chip integration of VCSELs with micro optical elements. Moreover, advancements in nanofabrication technologies, such as ultrafast laser three-dimensional (3D) printing technology^19–26^, have enabled the fabrication of various versatile micro optics, providing a more convenient way for photonic integrations. By combining the unique characteristics of VCSELs with the functionalities offered by functional optical elements, it enables various versatile integrated photonic devices and systems with compact size, low power consumption, high modulation bandwidth, and increased functionality.

In this review, we delve into the recent progress made in harnessing the capabilities of VCSELs for advanced integrated photonic devices and systems, highlighting their potential role in shaping the future of photonics. We start from the fundamentals of VCSELs and their advantages for forming integrated photonic devices and systems. Then, we discuss the operation principle, performance, and application of the state-of-art VCSEL-based integrated photonic devices and systems, including photonic neural networks, vortex beam emitters, holographic devices, beam deflectors, atomic sensors, and biosensors. At last, we conclude the paper and discuss the key challenges and future development direction of these integrated devices and systems. By shedding light on the latest developments, we aim to provide inspiration and guidance towards unlocking the full potential of VCSEL technology and its integration into advanced photonic solutions.

## Fundamentals, advantages, and advancements of VCSELs

### Structure and working principle of VCSELs

**Fig. 1:**
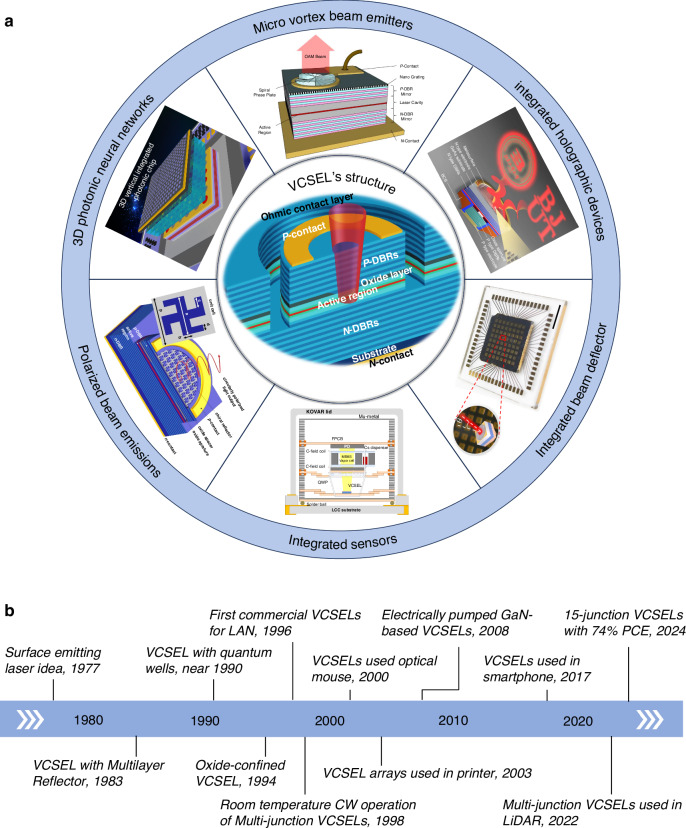
Overview of VCSELs-based integrated photonics. **a** Schematic diagram of a top-emitting VCSEL and integrated devices and systems based on VCSELs. **b** Timeline of VCSEL development

The central graph in Fig. 1a shows a schematic diagram of a top-emitting VCSEL, of which the epitaxial structures from bottom to top contain substrate, n-type distributed Bragg reflector (N-DBR) mirrors, active region, oxide layer, p-type DBR (P-DBR) mirrors, and Ohmic contact layer. The P-DBR and N-DBR mirrors consist of alternating layers of high- and low-index materials, typically formed by varying the mole fraction x in Al_x_Ga_1-x_As in GaAs-based VCSELs. For top-emitting configuration, the N-DBR mirrors have a high reflectivity close to 1.0, while the P-DBR mirrors have a lower reflectivity around 98.0–99.9% for light extraction. The DBR mirrors form the resonant cavity of VCSEL and provide optical feedback to the active region. The active region sandwiched by the P-DBR and N-DBR mirrors, generally consisting of multiple quantum wells, is responsible for generating light at a specific wavelength and providing optical amplification. To maximize the optical gain, the quantum wells in the active region are placed in the antinode of the standing wave. The oxide layer is formed by selective thermal oxidation of Al-rich AlGaAs layers, of which the Al component is generally higher than 0.98 and the thickness is typically 20–30 nm. The forming oxide aperture can provide excellent current confinement and optical confinement, leading to reduced threshold and increased power conversion efficiency. The oxide layer is generally placed in the node of the standing wave to optimize the far-field divergence. When the device is forward biased, the injected carriers are confined in the oxide aperture and undergo radiative recombination in the active region, and the generated photons experience multiple reflections within the resonant cavity and are amplified by the gain materials in the active region. When the threshold condition is reached, the amplified photons will radiate in a coherent manner, forming a monochromatic and coherent laser beam, of which the emission direction is perpendicular to the substrate.

### Advantages of VCSELs for integrated photonics

Compared with other lasers such as solid/gas lasers or EELs, VCSELs exhibit unparalleled advantages in size, power consumption, modulation bandwidth, and scalability^27,28^. In recent years, VCSELs open up a brand new path to realize compact, lightweight, and scalable advanced integrated photonic devices and systems, including 3D photonic neural networks, micro vortex beam emitters, polarized beam emitters, integrated holographic devices, integrated beam reflectors, and integrated sensors, as shown in Fig. 1a, which enriches their functionality and application. There are several unique characteristics and advantages that make VCSELs ideal laser sources for advanced integrated photonic devices and systems:

#### Wafer-normal emission and scalability

The wafer-normal, circular-beam emission of VCSELs is advantageous for 3D vertically stacked integration because it allows for easy alignment and straightforward efficient coupling with other optical components, facilitating compact and integrated photonic systems. Besides, it allows wafer-level manufacturing and testing for high-yield mass production, leading to lower cost. More importantly, the perpendicular emitting feature of VCSELs makes themselves can be fabricated into large scalable two-dimensional arrays, allowing for massive parallelism and high-density integration. By integrating multiple VCSELs with different wavelengths on a single chip through using patterned substrate^29^, grading spacer layer^30^, or intra-cavity grating with varying fill factor^31^, complex functions such as wavelength division multiplexing (WDM)^32,33^ can be implemented. This scalability is vital for applications where high-density integration is required.

#### High-speed direct modulation

VCSELs can be directly modulated at a very high speed, typically in the range of several gigahertz, due to their small volume. This allows for efficient high-speed data transmission without the need for external modulators. The direct modulation capability of VCSELs simplifies system design and reduces power consumption. More importantly, the modulation bandwidth of VCSELs can be greatly improved by proper epitaxial and structural design. Methods such as increasing the differential gain by adding strain in quantum wells^34–36^, decreasing the photon lifetime and the active area volume^37,38^, or reducing parasitic capacitance^39–41^ have been demonstrated efficient to increase the modulation bandwidth of VCSELs. Therefore, adopting VCSELs as the laser source can impart additional high-speed modulation characteristic to the integrated photonic devices or systems. By leveraging VCSELs’ inherent high-speed modulation characteristics, integrated photonic devices and systems can achieve increased data bandwidth, enhanced signal quality, and improved system performance, which is very important for applications such as optical computation and optical communication where high-speed data transfer is essential.

#### Low power consumption

Reducing the power consumption of the systems is one of the primary purposes of integration. With the advent of the big data era and the rapid development of AI computations like ChatGPT, energy consumption is an inevitable challenge to face. As the mainstream laser source for data center, VCSELs can achieve very low threshold currents at the sub-mA level^42–44^ and high power conversion efficiency larger than 50%^45,46^, which leads to relatively low power consumption. Additionally, high-speed VCSELs with energy-to-data rate ratio <100 fJ/bit have been reported^47,48^. This characteristic allows for lower energy consumption, reduced heat generation, and longer device lifetime.

#### Temperature stability

VCSELs exhibit better temperature stability compared to edge-emitting lasers^49–51^. Their emission wavelength shifts minimally with temperature variations, allowing for stable operation over a wide range of environmental conditions. Unlike EELs’ stronger dependence of gain-peak wavelength shift as a function of temperature, VCSELs operate only in a single longitudinal mode and their emission wavelength is highly stable against temperature and has minimal temperature dependence of optical cavity^8^. This attribute is crucial for applications requiring reliable performance in challenging thermal environments. In addition, the smaller wavelength shift of VCSELs allows using narrower-bandwidth filter in the receiver of detection systems like LiDAR, which is beneficial for obtaining a higher signal-to-noise ratio.

### Advancements of VCSELs

Figure 1b shows the timeline of VCSEL development. Since the idea of surface emitting laser (SEL) was proposed by Professor Iga in 1977, VCSELs have attracted a great deal of interest and extensive research in the past four decades. The first SEL was experimentally realized by Professor Iga group in 1979^52^. After that, various methods, including using semiconductor distributed Bragg reflectors (DBRs) as mirrors^53^, introducing quantum wells in active region^54–56^, and implementing oxide apertures for current confinement^57^, have been demonstrated to evolve and improve the device we now call a VCSEL. Due to the significant and pioneering contributions of all researchers in this field, VCSELs have made great progress in material growth, device structure, fabrication process, and device performance. So far, VCSELs have been widely used in optical communication, optical mouse, laser printer, optical storage, and consumer electronics such as smartphones, vehicle LiDAR, AR/VR, etc.

**Fig. 2:**
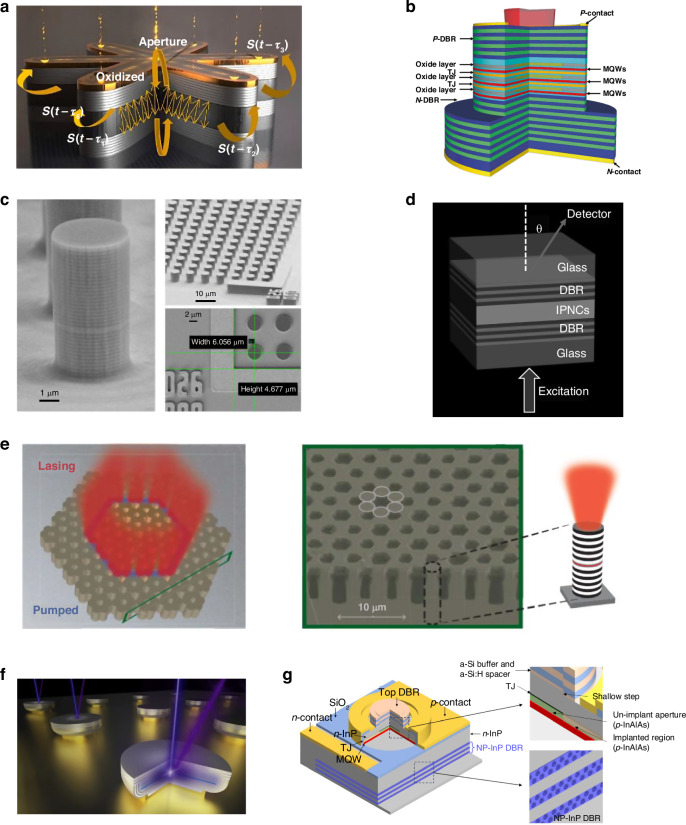
VCSELs with novel designs. **a** Novel VCSEL design of a multiple transverse-coupled-cavity for achieving high modulation bandwidth beyond 45 GHz^58,59^. **b** Multi-junction configuration of VCSEL for boosting the output power^70^. **c** VCSEL using perovskite as the gain material in active region^72^. **d** Micropillar VCSEL array^75^. **e** Topological insulator VCSEL array^76^. **f** GaN-based UVB VCSEL^82^. **g** InP-based VCSEL with nanoporous DBRs^87^. Figure **a** is reprinted from ref. ^59^ with permission from the De Gruyter by CC BY 4.0. Figure **b** is reprinted from ref. ^70^ with permission from the Springer Nature by CC BY 4.0. Figure **c** is reprinted from ref. ^72^ with permission from the American Chemical Society, Figure **d** is reprinted from ref. ^75^ by CC BY 4.0. Figure **e** is reprinted from ref. ^76^ with permission from the American Association for the Advancement, figure **f** is reprinted from ref. ^82^ with permission from the American Chemical Society by CC BY 4.0, and figure **g** is reprinted from ref. ^87^ with permission from the Optica Publishing Group

In recent years, VCSELs with new structures and new materials have been continuously developed to further improve their performance. Dalir et al. recently proposed a novel VCSEL design of a multiple transverse-coupled-cavity (MTCC) coupled through a central cavity^58^, of which the structural schematic diagram of the VCSEL is shown in Fig. 2a. The MTCC can provide slow light optical feedback into the modulation cavity, and thus extends the VCSEL bandwidth beyond the limit of relaxation oscillation frequency. The VCSEL demonstrates a state-of-art modulation bandwidth of 45 GHz and can be further increased to beyond 100 GHz by optimizing the device structure^59^, which is unparalleled by other lasers.

To boost the output power of VCSELs, multi-junction VCSELs have been proposed^60–63^ and rapidly developed in recent years^64–71^. Figure 2b shows a schematic diagram of a typical three-junction VCSEL. The multi-active regions connected via tunnel junction can greatly improve the roundtrip gain in the cavity, thus boost the output power and the differential quantum efficiency. Recently, 15-junction VCSELs with a recorded power conversion efficiency of 74% at room temperature under nanosecond driving current has been realized^70^, the corresponding differential quantum efficiency exceeds 1100%. To reduce the large divergence of multi-junction VCSELs caused by the strong optical guiding introduced by multi-oxide layers, Zhang et al. introduced a novel antireflective light reservoir into multi-junction VCSELs and achieved a small full divergence angle of 8.0^o^ and a recorded single-mode output power of 28.4 mW^71^. In addition to new device structures, new gain materials such as perovskite were tried to serve as the gain medium materials in the active region^72–74^, as shown in Fig. 2c. Compared with traditional semiconductor gain materials, perovskite materials can be solution-processed easily without the need of high-temperature heating or epitaxy, and their optical bandgaps can be tuned in the visible to infrared regions, which is attractive for low-cost large-area optoelectrical applications. But how to achieve monolithic epitaxial growth of the DBRs structure and realize electrically injected perovskite-based VCSELs is still challenging.

In recent years, driven by applications such as neuromorphic computing and topological photonics, some new types of VCSEL arrays have been proposed, such as micropillar VCSEL arrays for reservoir computing shown in Fig. 2d^75^ and topological insulator VCSEL array shown in Fig. 2e^76^, respectively. These arrays exhibit excellent characteristics such as high spectral homogeneity and high coherence, which hold significant potential in applications varying from neuromorphic computing to optical communication. Although optically pumped topological insulator lasers have witnessed fast advancements, electrically pumped topological insulator lasers are currently in their early stages of development. The hurdle lies in designing a topological structure that simultaneously provides effective carrier injection and large mode confinement^77^.

GaN-VCSELs and InP-based VCSELs have also made great progress in recent years. Unlike GaAs-based VCSELs, the material systems of GaN-based VCSELs and InP-based VCSELs do not support a high index contrast and have a larger lattice mismatch, making it challenging to grow high-quality DBRs. However, considerable efforts have been made to circumvent the challenge. Different cavity structures, including double dielectric DBR cavities, hybrid DBR cavities, and fully epitaxial DBRs have been developed for GaN-VCSELs^78–81^. So far, electrically injected GaN-based VCSELs covering 405–565 nm have been realized^81^, but the development of ultraviolet (UV) VCSELs operating below the 400 nm wavelength presents a more complex challenge and electrically injected UV VCSELs within the sub-400 nm region are yet to be achieved. Key obstacles encompass the high-gain active layer growth in the UV range, the creation of a low-loss microcavity, and the achievement of efficient current injection mechanisms. Recently, optically pumped UV VCSELs in the UVB spectrum (280–320 nm, Fig. 2e) and the UVC spectrum (200–280 nm) were realized^82,83^. The future work is to find solutions for developing electrically injected UV VCSELs.

In terms of InP-based VCSELs, numerous strategies have been devised to address the obstacles posed by heteroepitaxial DBRs on InP. These methods encompass growing long-wavelength active regions on GaAs substrates (such as dilute nitrides^84^), dual-dielectric DBRs^85^, and wafer fusion of InP active regions with AlGaAs DBRs^86^. More recently, Li et al. provided an alternative pathway of creating highly uniform, monolithic, and homoepitaxial DBRs on InP substrates (Fig. 2f)^87^. By manipulating the index contrast through photonic nanostructures formed by electrochemical porosification, alternating nanoporous (NP) and nonporous InP layers with a record index contrast near 1.0 and near-unity reflection of DBRs with around 10 pairs can be achieved, and VCSELs with 1380 nm and 1550 nm wavelengths have been realized, which are attractive laser sources for silicon photonics and long-reach data communications.

## Advanced integrated photonic devices and systems based on VCSELs

### VCSEL-based micro vortex beam emitters

Vortex beams have a helical wavefront and naturally carry orbital angular momentum (OAM), so vortex beams are also known as OAM beams, of which the complex amplitude expressions have a helical phase term exp(*ilφ*), where *l* is the topological charges of the OAM state and *φ* is the azimuthal angle^88^. OAM can be utilized as a new degree of freedom for information encoding. Due to the orthogonality and infinite topological charge number of OAM states^89,90^, vortex beams can be used to greatly improve the capacity and spectral efficiency of optical communication systems^91–93^. Apart from large-capacity optical communication, vortex beams also exhibit enormous potential in myriad applications, including quantum information^94–96^, particle manipulation^97,98^, holography^99–103^, optical data storage^104,105^, and optical encryption^106^.

**Fig. 3:**
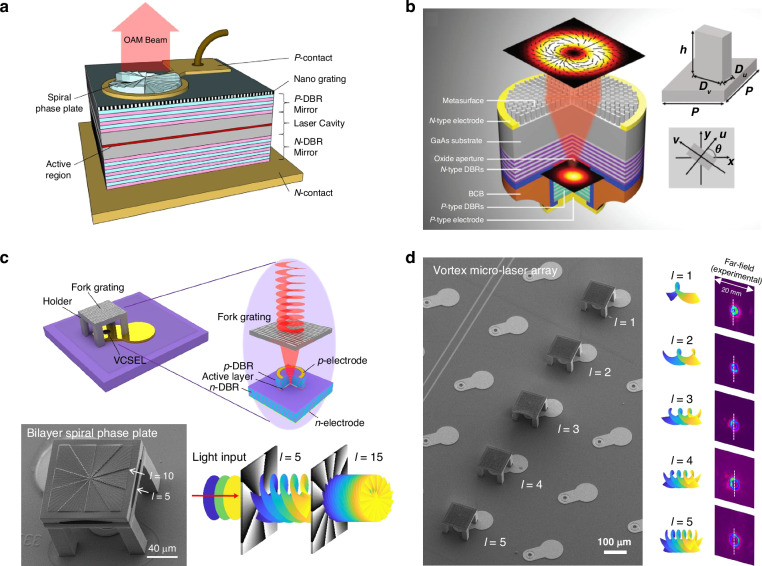
Micro vortex beam emitters based on VCSELs. **a** Schematic of the VCSEL with an integrated spiral phase plate on the emitting surface for direct OAM beam generation^108^. **b** Schematic illustration of the VCSEL with integrated metasurface to create complex vectorial fields with well-defined wavefronts, the inset shows the employed meta-atom with rectangular cross-section^114^. **c** Top row: schematic illustration of the VCSEL with an integrated non-contact spiral phase plate fabricated by fs laser 3D printing^115^. Bottom row: Scanning electron microscope (SEM) image of a fabricated VCSEL integrated with two-layer cascaded SPPs for generating vortex beam with *l* = 15. **d** Addressable and scalable 1 × 5 vortex micro laser array with specific topological charge in each channel^115^. Figure **a** from ref. ^108^. with permission from the Optica Publishing Group. Figure **b** is reprinted from ref. ^114^. with permission from the John Wiley and Sons. Figures **c** and **d** are adapted from ref. ^115^. with permission from the American Chemical Society

Compared with the bulky optical systems composed of cascaded optical elements for generation of vortex beams, integrated chip-scale vortex beam emitters offer a more compact and robust solution. Monolithic OAM laser has been realized by integrating a DFB laser with a ring vortex emitter where the OAM beam can emit vertically^107^. However, the threshold of the laser is larger than 50 mA and the optical output power is lower than 0.4 mW, resulting in a relatively high power consumption. In contrast, VCSELs offer a much more energy-efficiency manner to realize chip-scale OAM emitters. By utilizing the feature of wafer-normal emission of VCSELs, micro spiral phase plates (SPPs) can be integrated directly on the emission surface of VCSELs, as shown in Fig. 3a^108^. The circularly symmetric beam profile of VCSELs can uniformly illuminate into the integrated SPPs, which makes it easy to generate structured light and OAM beams. Spiral phase shift sectors with a 0–2π phase variation are fabricated well atop the VCSEL emitting aperture, and the total phase shift of 2π is divided into eight equally spaced discrete levels within each phase shift sector. The SPPs were fabricated in a 1000 nm thick silicon nitride film deposited on the top of the VCSEL and patterned using a focused ion beam (FIB) etching technique. The integrated device can generate high-purity OAM modes and their superposition states. The threshold current and maximum output power of the device is 1 mA and 4 mW, respectively, showing great improvement than the DFB laser-based OAM solution. Similarly, micro dielectric axicon can be integrated with VCSEL by FIB etching technique to realize Bessel beams^109^. This method relies on controlling the optical path of light to manipulate the phase retardance of the emitting beam from VCSEL, which cannot control the polarization states.

Metasurface consisting of 2D array of birefringent nano-antennas enables an ultracompact and powerful solution to control both phase and polarization properties of the light^110–112^. Compared with conventional diffractive optical elements (DOEs), metasurface is a kind of two-dimensional optical component with planar configuration. The distinctive flat design, potential for large-scale integration, and compatibility with complementary metal-oxide-semiconductor processing make metasurfaces highly suited for optoelectronic integration. Besides, metasurfaces have a more powerful light-field modulation capability. For example, the plasmonic metasurfaces based on metal nanorods enable the multiplexed modulation of multiple physical dimensions of light including polarization, OAM, and wavelength^113^. Fu et al. proposed a chip-scale OAM emitter achieved by integrating metasurface on the back side GaAs substrate of a bottom emitting VCSEL^114^, as shown in Fig. 3b. The GaAs nano-pillars with rectangular cross-sections can be considered as truncated waveguide with anisotropic effective refractive indices of the waveguide modes polarized along their fast and slow axis (see the inset in Fig. 3b). Consequently, independent control of the propagation phase imposed to the orthogonally polarized light along these two axes can be achieved by changing its width (*D*_*u*_) and length (*D*_*v*_), respectively, which can be leveraged to manipulate both the local polarization states and the phase retardance. By controlling the phase propagation, phase offset along the two orthogonal directions of the metasurface elements, and their orientation angles, the linearly polarized beam of the VCSEL can be easily transformed into any complex wavefront with desired phase profiles and polarization states. By integrating such a kind of metasurface with VCSELs, vector vortex beams with different polarization orders (m = 1, 2, and 3) have been realized.

However, the current VCSEL-based vortex lasers have difficulty in realizing large topological charges larger than *l* = 5 due to the insufficient space-bandwidth product (SBP) caused by the inherent limited emitting area of VCSELs. This will limit the information capacity of OAM-based multiplexing applications. To address this issue, cascaded SPPs are designed and directly integrating on top-emitting VCSEL by using fs laser 3D printing technique^115^, as illustrated in Fig. 3c. The printed phase plates are integrated with VCSELs through a non-contact way, which can minimize the impact of the phase plates on the VCSELs’ inherent characteristics. To achieve large topological charges, two SPPs with *l* = 5 and *l* = 10 are cascaded to generate a vortex beam for *l* = 15. And larger topological charges were also proven to be feasible by increasing the phase plates. This breaks the limit of the topological charges of current vortex microlasers and may unlock the on-chip application of OAM-based information multiplexing with more channels. In addition, the VCSEL shows a modulation bandwidth larger than 11 GHz, indicting high-bit-rate data can be imprinted on the OAM beam by directly modulating the high-speed VCSEL, which may further increase the capacity of on-chip information multiplexing communication. More importantly, addressable and scalable vortex micro laser array with specific topological charge in each channel can be realized by harnessing the scalability of VCSELs, as is proved by a 1×5 addressable vortex laser array shown in Fig. 3d. These capabilities hold great promise for applications ranging from high-capacity optical communications to data storage.

### VCSEL-based integrated holographic devices

Holography has revolutionized imaging and display technologies by enabling the reconstruction of three-dimensional scenes with stunning realism, which provides realistic and immersive visual experiences^116–118^. Traditional two-dimensional images lack depth and fail to convey the full spatial information of an object. Holography addresses this limitation by capturing and reproducing the complete wavefront of light, resulting in lifelike three-dimensional representations. Traditional spatial light modulator (SLM) based holography methods can achieve dynamic and reconfigurable holograms. However, the large size of SLMs inevitably increasing the optical system size and makes it difficult to achieve integration with VCSELs^119^. In addition, the large pixel size, low resolution, small field-of-view (FOV), limited space-bandwidth, and unwanted diffraction orders of SLM-based holography restrict the possibility of improving the quality of reconstructed images^120,121^.

**Fig. 4:**
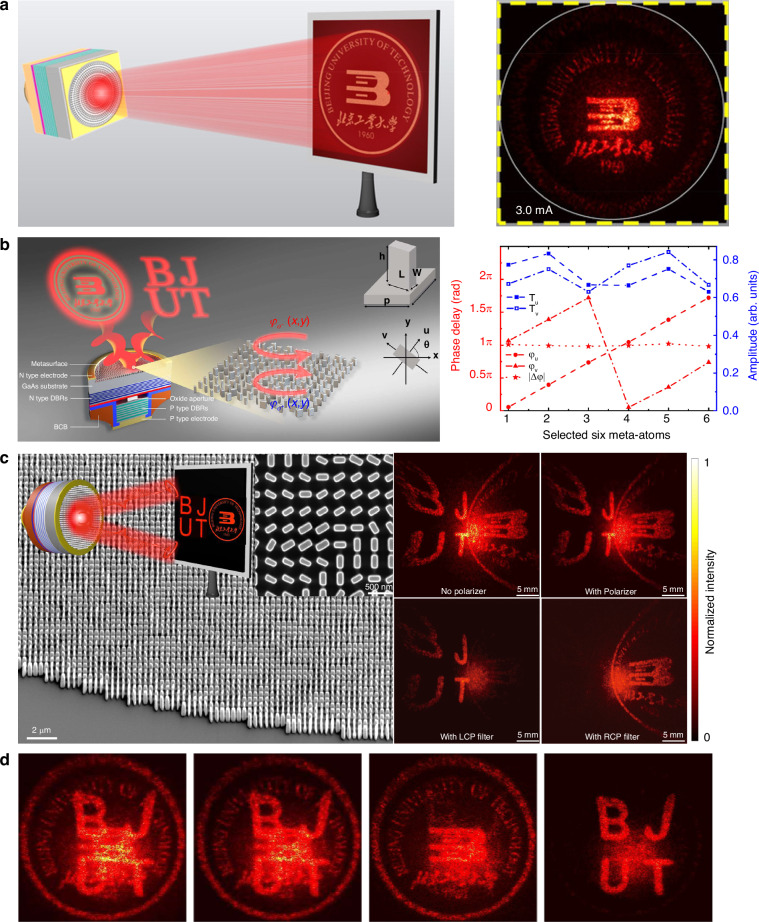
Integrated holographic devices based on VCSELs. **a** Left: schematic illustration of a holographic VCSEL integrated with metasurface for constructing a university logo image. Right: Experimentally reconstructed image^122^. **b** Left: polarization multiplexed holography to display two different holographic images in the far field. Right: calculated transmission amplitude and phase of the selected six meta-atoms. A complete 2π modulation range of propagation phase is achieved^124^. **c** Left: SEM images of the integrated holographic metasurface. The inset schematically summarizes the example of dual-channel holographic design to structure the far-field intensity profiles of the polarization-decoupled VCSEL into two channels, in which two infrared images of the letters of “BJUT” and the university logo were directly projected onto a white screen for observation^124^. Right: the recorded far-field beam patterns without and with a polarizer, in which the uncontrolled co-polarization channel can be filtered out using a polarizer. **d** The divergent co-polarization beam spot can be removed from the holographic images with a polarizer, revealing the hidden information^124^. Figure **a** is adapted from ref. ^122^ with permission from the John Wiley and Sons. Figure **b**–**d** are adapted from ref. ^124^. with permission from the Springer Nature by CC BY 4.0

VCSELs provide a perfect integration platform for miniaturizing holographic devices. With VCSELs as coherent light sources, holographic displays can be realized with improved performance and reduced complexity. Wang et al. proposed an integrated holographic device based on VCSELs^122^. By integrating VCSELs with metasurface holograms that can be encoded with arbitrary complex beam patterns, chip-level holographic devices have been realized. Hsu. et al. extended this method to photonic crystal surface-emitting lasers (PCSELs) and demonstrated a reconstruction of holographic images by integrating metasurface holograms with PCSELs^123^. The left column in Fig. 4a illustrates the schematic of a chip-level VCSEL-based holographic device. A phase-only metasurface hologram with a lattice size of 260 nm is designed using the classical Gerchberg–Saxton algorithm to construct an image of the university logo in the far field of the VCSEL. The right column in Fig. 4a shows a measured holographic image projected onto a screen placed at *Z* = 1 cm under an injection current of 3 mA. Thanks to the subwavelength lattice constant of the metasurface hologram, high efficiency and large FOV of 124° were realized. Besides, the holographic image is free of the zeroth-order laser mostly due to the divergence of the incoming wave, which is beneficial for a simple design and a high signal-to-noise ratio. It should be noted that once the metasurface structure is designed and fabricated, the generated hologram is usually fixed, which is still challenging in achieving dynamic and reconfigurable holograms. Therefore, developing tunable metasurfaces is a direction to circumvent this challenge. Alternatively, micro-electro-mechanical systems (MEMS) can be considered to integrate on the VCSELs to achieve dynamic modulation. In addition, by integrating metasurface consisting of 2D array of birefringent nano-antennas with VCSELs, polarization multiplexed holography can be realized. Ni et al. utilized Jones matrix metasurfaces composed of birefringent nano-fin arrays to monolithically integrate with bottom-emitting 980 nm VCSELs for on-chip decouple the circularly polarized (CP) states of the laser beam and modulate the phase profiles of each spin component independently to realize dual-channel holographic images projection^124^, as shown in the left column in Fig. 4b. The VCSEL substrate is with a large thickness of 630 μm to expand the laser beam, thus to ensure sufficient diffraction through the interaction with the most part of nanostructures forming the integrated metasurfaces. A basic rectangular pillar of the metasurfaces can be used as a truncated waveguide that controls the propagation phases of two eigenmodes with different polarizations. By adjusting the width (*W*) and length (*L*) of the pillar, the propagation phases for the fast and slow axes can be independently controlled, as illustrated in the inset of Fig. 4b. To achieve a full 2π modulation of propagation phase, six nanopillars numbered 1–6 were employed, each providing an equivalent phase step of π/3. The phase difference between the two eigen-polarization channels remained fixed at π, as shown in the right column of Fig. 4b. The wavefronts of both the output right-circularly polarized (RCP) and left-circularly polarized (LCP) components were holographically structured to display two different far-field images, and each component can be filtered out with a CP polarization filter, as shown in Fig. 4c. More importantly, the orthogonality of the VCSEL spin states can be utilized to further encrypt the images with polarization selectivity. By designing the CP components of the holographic VCSEL to travel in the same direction, their beam patterns are allowed to mix. This mixture enables access to distinct holographic information separately by utilizing CP filtering, as illustrated in Fig. 4d. Such multiplexing techniques are highly desired to optimize the tremendous information capability and improve the space-bandwidth product of metasurface holograms. Since a polarization multiplexed holography can carry high-dimensional information rather than the simple scalar intensity information, combining polarization multiplexed holography with VCSELs allows to fully exploit their polarization degree of freedom as an additional information channel, which could substantially boost the capacity of VCSELs in optical communication, display, data storage, and optical encryption, etc.

### VCSEL-based integrated beam deflector

Beam deflectors are essential components in numerous applications, including LiDAR, projection displays, and free-space communication^125–127^. Traditional mechanical beam deflectors are often bulky and suffer from mechanical wear due to the moving parts. In contrast, nonmechanical beam-steering techniques eliminate the need for mechanical components, reducing the risk of wear, mechanical failures, and the associated maintenance requirements, which offer a more reliable, flexible, and compact beam-steering solution. In recent years, tremendous nonmechanical beam-steering techniques based on an optical phased array (OPA)^128–130^, SLM^131,132^, etc., have been demonstrated, yet additional off-chip laser sources are needed.

VCSELs provide an excellent platform for implementing beam steering due to their inherent easy monolithic integration and fast modulation capabilities. By on-chip integration of phase control elements with VCSELs, precise control and steering of the emitted beam can be achieved in a compact and efficient manner. One direct beam steering method is to adjust the phase difference between neighbor elements of an addressable coherently coupled VCSEL arrays through asymmetry current injection^133,134^. By varying the injection current, the refractive index of the semiconductor materials changes due to the combined effect of carriers and thermal effect, thus the phase difference between the emitters varies and finally beam steering happens. This method does not require additional integrated optical elements, but the output power and emitted wavelength are unstable due to the varied emitted modes caused by varied injection currents.

**Fig. 5:**
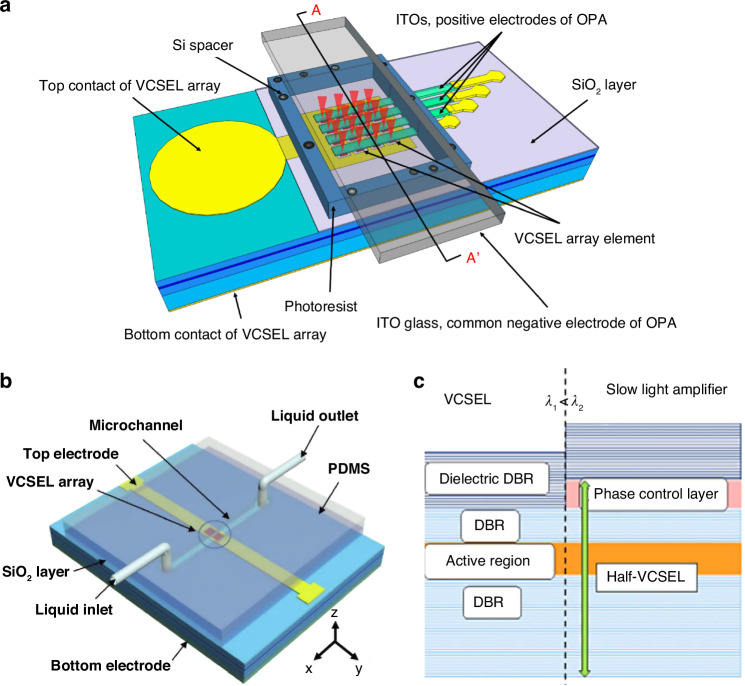
Integrated beam deflector based on VCSELs. **a** Schematic illustration of a coherent VCSEL array integrated with OPA for beam steering^135^. **b** Schematic illustration of the integration of microfluidic channel with VCSEL to control the laser beam^136^. **c** Schematic of a beam scanner based on the integration of VCSEL with slow light waveguide^137^. Figure **a** and **b** are reprinted from refs. ^135^. and ^136^. respectively with permission from the Optica Publishing Group. Figure **c** is reprinted from ref. ^137^. with permission from the Chinese Laser Press

To address this issue, Pan et al. proposed a nonmechanical beam steering device composed of a coherently coupled VCSEL array and a liquid crystal OPA^135^, as illustrated in Fig. 5a. Since there is a fixed phase difference between the emitters in the coherently coupled VCSEL array, dynamic beam steering can be achieved by tuning the phase difference through adjusting the voltages loaded on the liquid crystal OPA. The OPA has less impact on the stability of the VCSEL array’s output power and wavelength due to the modulation of the laser beam is implemented outside the VCSEL cavity. Zhao et al. proposed a beam deflector achieved by integrating microfluidic channel on a coherently coupled VCSEL array^136^, as shown in Fig. 5b. Dynamic beam steering can be achieved by the injection of liquids with different refractive index into the microchannel. These two devices provide solutions to implement a compact laser system with real-time beam steering, but at the cost of limited steering range within 6.06°and slow response time larger than 500 ms due to the inherent properties of liquid crystal and the slow exchange of liquids.

Hu et al. proposed a VCSEL-based beam steering device with large steering angle and high steering speed^137^, as shown in Fig. 5c. The monolithic beam steering device is realized by laterally integrating a VCSEL with a slow-light waveguide (amplifier) which has a longer wavelength. The slow-light waveguide can enable large dispersion, thus large steering range with high resolution can be achieved by tuning the input wavelength or tuning the refractive index of the slow-light waveguide material^138–141^. By continuously changing the injection current of the VCSEL, the lasing wavelength varies continuously due to the self-heating effect, and then the slow light coupled from the VCSEL is amplified in the waveguide and continuous sweep of the beam deflection angle is achieved. At the same time, beam steering can be realized by changing the refractive index of the amplifier. The 3 dB bandwidth of beam steering is over 70 kHz, which is much faster than that of the liquid crystal OPA-integrated beam steering devices.

**Fig. 6:**
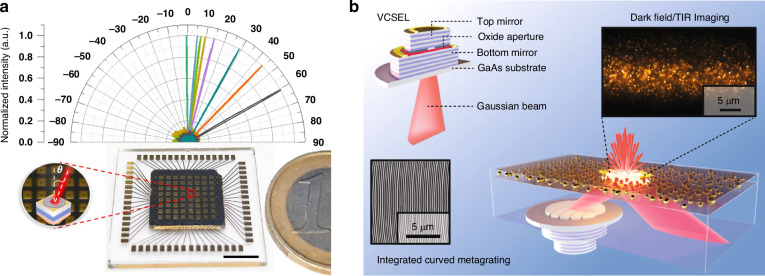
Integrated beam deflector achieved by integrating VCSELs with metasurfaces. **a** On-chip 2D metasurface-integrated 8 × 8 VCSEL array allows for programmable beam steering with large deflection angle ranging from 0° to 60° (ref. ^122^). **b** Schematic illustration of an oxide-confined bottom-emitting GaAs-VCSEL and its integration with a metagrating that enables high-angle deflection for high-contrast dark field and total internal reflection microscopy^142^. Figure **a** is reprinted from ref. ^122^. with permission from the John Wiley and Sons, Figure **b** is reprinted from ref. ^142^. with permission from the Springer Nature by CC BY 4.0

An alternative method to realize chip-scale beam reflector is to integrate VCSEL with metasurface. Wang et al. demonstrated an on-chip 2D metasurface-integrated 8×8 VCSEL array allows for programmable beam steering with a large deflection angle ranging from 0° to 60°^122^, as shown in Fig. 6a. Each metasurface on the chip is designed with a different phase profile for different deflection angle. The fabricated beam steering chip has a small size comparable with a coin. This large-angle beam steering chip is beneficial for high-speed applications and the generation of wide-field spatially structured light. Juodėnas et al. demonstrated the integration of a novel axicon formed by curved GaAs metagratings with a VCSEL to enable high-angle and high-efficiency deflection^142^, as shown in Fig. 6b. The unique axicon design that achieves both high-angle deflection and quasi-collimation by utilizing the curvature of the metagrating. The design effectively aligns the tangential hyperbolic phase gradient with the input phase generated by the VCSEL through an offset distance. The integration of metagrating-based VCSELs as illumination sources in microscopy demonstrates impressive switchable dark-field and total internal reflection (TIR) illumination capabilities. Compared to advanced illumination devices, this solution offers the advantages of affordability, efficiency, compactness, and a built-in, pre-aligned light-coupling system.

### VCSEL-based integrated sensors

The characteristics of VCSELs, such as low power consumption, circular beam emission, small size and high temperature stability, make them quickly become the mainstream light source for various sensing applications. Typical sensing applications, including mice, gesture recognition and face recognition in smartphones, LiDAR for autonomous vehicle, have been already demonstrated detailly^7,57^. Here, we focus on miniaturized VCSEL-based biosensor and atomic sensors.

#### Biosensors based on VCSELs

Small portable miniaturized total analysis systems based on microchannels or microarrays hold the promise of providing immediate point-of-care services that could facilitate the detection of common diseases and early cancers^143^. In addition, integrated bioanalysis systems can provide a parallel architecture for high-throughput experiments such as drug screening and enable scientists to better understand complex biological processes, such as protein interactions with potential drug targets. More importantly, the development of miniaturized analysis systems will improve and enable in flexible diagnostic tools such as imaging and real-time drug delivery systems. Fluorescence sensing remains one of the most widely used techniques in biotechnology. Unfortunately, traditional bioluminescence sensing systems use bulky discrete components that are expensive and require a large footprint and precise alignment^144^. The integrated on-chip sensing architecture makes portable and powerful medical diagnostic devices practical and is expected to reduce the cost of instruments.

**Fig. 7:**
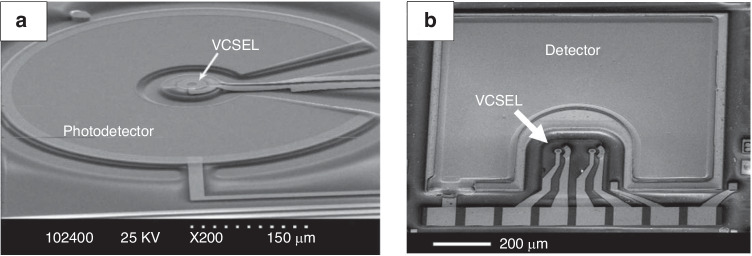
SEM micrographs of monolithically integrated biosensors based on VCSELs ^143,147^. **a** is reprinted from ref. ^143^. with permission from the IEEE. **b** is reprinted from ref. ^147^ with permission from the Optica Publishing Group

The field of biosensing is increasingly relying on integrated photonics for label-free and highly sensitive detection of various biological analytes. VCSELs have unique advantages in this field due to their small size, low power consumption, superior wavelength stability^145^. More importantly, the narrow linewidth of single-mode VCSELs is generally less than 0.2 nm, which can generate sharp excitation of the desired fluorescent probe and maximize its spectral separation from the emission peak for more efficient spectral filtering and thus improve fluorescence sensitivity and resolution^146^. VCSELs can enable highly integrated biosensors with a size of 100 μm-level^143,147^, as shown in Fig. 7a, b. Integrating VCSEL, photodetector, and filter on the same substrate in a single piece can dramatically reduce the cost and size, and increase functionality and sensitivity of the biosensors. With the monolithic approach, the detection channel spacing or pixel spacing can be reduced to less than 100 μm, which is challenging in discrete component systems. The sensor units can be manufactured in parallel using traditional semiconductor manufacturing techniques and used as the building blocks of highly parallel detection systems.

**Fig. 8:**
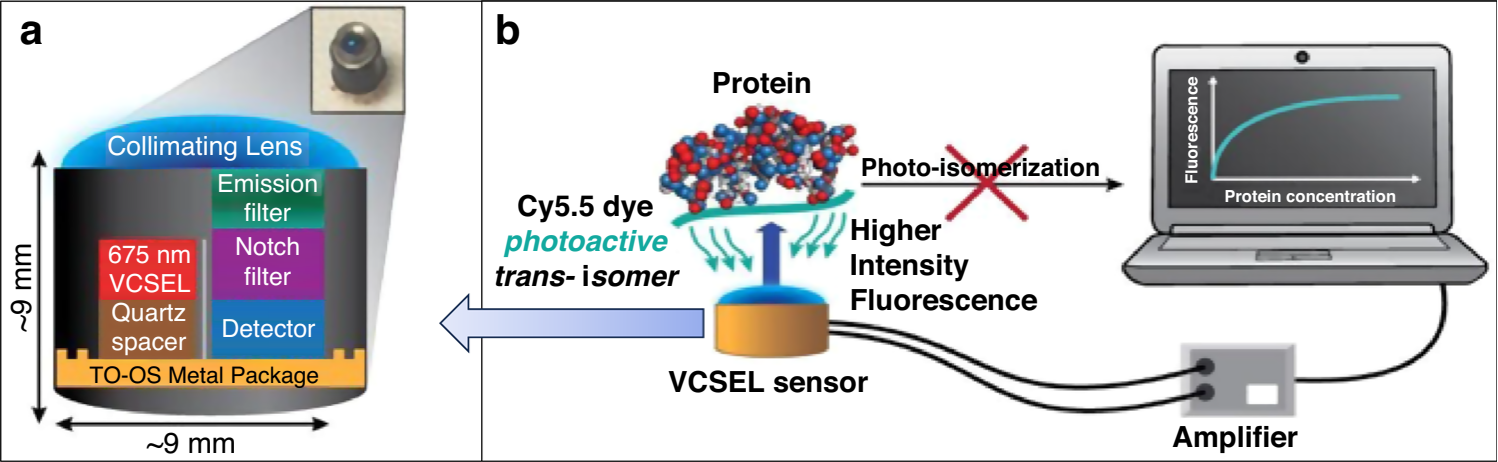
VCSEL-based biosensor with small-footprint TO package. **a** Cross-sectional view of the biosensor showing the optical components - VCSEL, PIN photodetector, and optical filters^146,148^. **b** Protein-induced fluorescence enhancement of a cyanine dye^146,148^. Figures **a** and **b** are reproduced from ref. ^148^. with permission from the Elsevier

Despite the potential for extreme miniaturization, monolithic designs suffer from performance limitations due to interlayer and lateral optical crosstalk (spontaneous emission) between the laser and the photodetector^146^. To address this issue while still maintaining a small device footprint, Mahzabeen et al. proposed a solution involves discreetly integrating separate VCSELs and detector components into a packaged sensor^146,148^, as shown in Fig. 8a. The biosensor consists of a 675 nm VCSEL and a custom PIN GaAs detector wire bonded to a TO-5 metal can package, which is designed to excite and record fluorescence in the near-infrared region. The VCSEL is placed on a quartz spacer for galvanic isolation and spatial separation from the detector, reducing the optical crosstalk, which is further mitigated by a black Ti/Au baffle. Placing the VCSEL and detector in a coplanar arrangement reduces background interference and increases the detection limit compared to the arrangement where the excitation light is incident on the detector. At the top of the detector is an integrated dielectric notch filter combined with a hybrid-bonded fluorescence emission filter that contains a 2 mm thick layer of RG695 absorbing glass for maximum rejection of excitation light. The multilayer filter emits through a positive incidence fluorescence centered at 750 nm with a bandwidth of 40 nm and rejects at least 6 orders of magnitude of the 675 nm excitation. The packaging sidewalls are painted black to reduce stray light reflections. The integration completes the installation of an anti-reflective collimating lens. The whole sensor package is only 9 mm in height and diameter, exhibiting a relatively small footprint. The authors used protein-induced fluorescence enhancement of a near-infrared cyanine dye to achieve instantaneous detection of protein in serum or urine samples. The non-covalent interaction between the dye and protein leads to the stabilization of the trans conformation in the photoactive state. This stabilization effect causes the excited-state lifetime to increase and enhances its quantum yield. When the VCSEL laser excites the sample, the enhanced emitted fluorescence is captured by the hybrid-integrated photodetector. The detected fluorescence generates an electric current that directly correlates with the concentration of protein present in the sample, as illustrated in Fig. 8b. This biosensor exhibits exceptional sensitivity (urine LOD = 0.023 g/L, LOQ = 0.075 g/L) and can accurately detect protein levels across a wide range of concentrations relevant to the human body. When compared to standard clinical assays and fluorimetry tools, this sensor consistently delivers precise quantification of total protein in urine samples from diabetic patients. Additionally, the VCSEL biosensor’s compatibility with miniaturized electronics makes it suitable for integration into a portable, cost-effective, user-friendly device that can provide sensitive and real-time measurements of total protein from small biofluid samples.

#### Atomic sensors based on VCSELs

Atomic sensors such as atomic clocks, magnetometers, and gyroscope, relying on the interaction between laser and atoms to achieve precise measurements of time, magnetic field, and attitude, play a crucial role in various scientific and industrial applications due to their exceptional accuracy and sensitivity^149,150^. One of the major trends for atomic sensors is the volume reduction that facilitates the integration into compact electronic devices and systems.

**Fig. 9:**
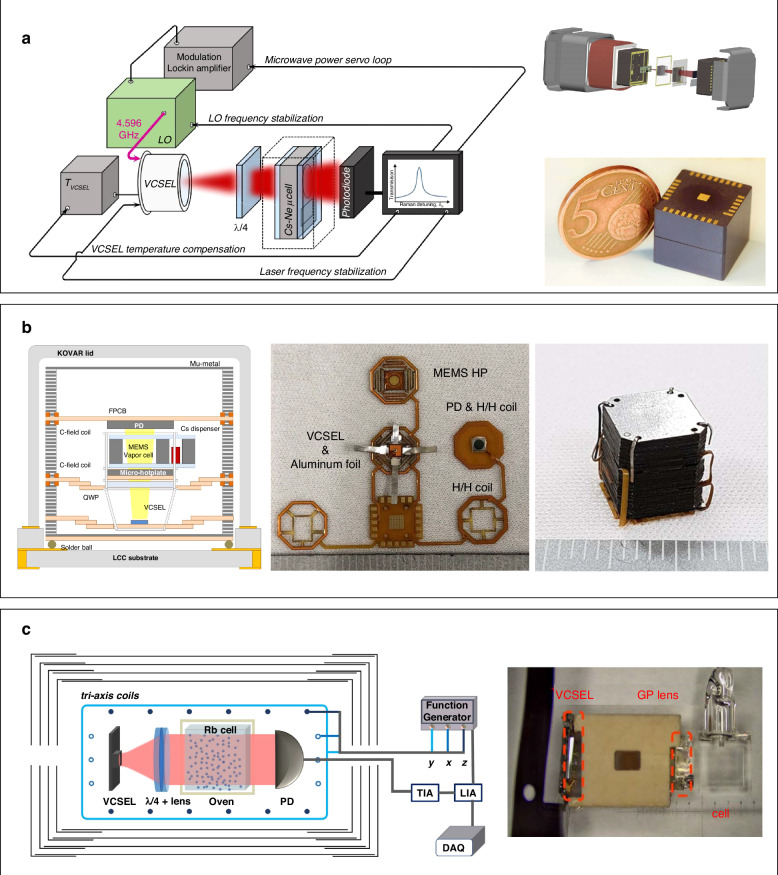
VCSEL-based miniaturized atomic sensors. **a** Architecture of a clock system with additional stabilization loops on the mid- and long-term stability. The clock physics package is fully integrated in a 15 × 15 × 13 mm^3^ cube^157^. **b** The cross-sectional view, the expanded view, and physics package of a chip-scale atomic clock with 3D integrated physics package^158^. **c** Left: configuration of single-beam miniature atomic magnetometer applying VCSEL^159^; FG function generator, TIA transimpedance amplifier, LIA lock-in amplifier, DAQ data acquisition. Right: photograph of VCSEL pump laser, GP lens, and vapor cell. Figures **a**–**c** are reproduced from refs. ^157–159^, respectively with permission from the IEEE

Chip-scale atomic sensors are highly attractive due to their advantages of small size, low power consumption, high precision and sensitivity, and excellent stability and long-term reliability^151^. However, conventional bulk lasers are not suitable for achieving chip-scale atomic sensors. In contrast, VCSELs with single mode, narrow linewidth, and stable polarization for atomic sensing have been demonstrated^152–154^ and enabled small low-power atomic sensors, including atomic clocks, magnetometers, and gyroscopes^155,156^. Vicarini et al. proposed an 895 nm VCSEL-based (tuned on the Cs D1 line) miniature atomic clocks based on coherent population trapping with improved fractional frequency stability of 7.5 × 10^−11^ at 1 s and better than 2 × 10^−11^ at 1 day^157^. The improved mid- and long-term frequency stability benefits from the implementation of additional stabilization loops, including a loop for stabilizing the actual temperature of the VCSEL chip, and another loop for maintaining the total microwave power absorbed by the laser to a value that maximizes the optical absorption and significantly reduces the laser power dependence of the clock frequency (Figure 9a left column). The implementation of these additional stabilization loops can reduce temperature-induced light-shift effects and lead to improved clock frequency stability. The clock system is packaged into a cube with small external dimensions of 15 mm ×15 mm × 13 mm, which is comparable with a five EURO cent coin.

Park et al. proposed a flexible hybrid approach for a 3D integrated physics package of chip-scale atomic clocks^158^. The cross-sectional view, the expanded view, and physics package of the chip-scale atomic clock are shown in Figure 9b. The package consists of a vertically stacked configuration incorporating a VCSEL, a MEMS hotplate, a quarter wavelength plate (QWP), and an alkali MEMS vapor cell, all bound together using aluminum foil. The aluminum-bound structure is suspended by the polyimide core of the flexible circuit board (FPCB) to ensure high thermal resistance. Additionally, a mu-metal fixture is utilized for magnetic shielding and FPCB suspension, while the MEMS hotplate and thermistor facilitate effective thermal management. The planar integrated FPCB is folded and stacked at an intersection with the mu-metal fixture. These innovations enable the chip-scale atomic clock with the proposed physics package to achieve a coherent population trapping (CPT) linewidth of 5.6 kHz and consume approximately 200 mW of power. The measured frequency short-term stability at 1000-second integration is around 5.2 × 10^−12^. These encouraging results demonstrate the promising performance of the flexible hybrid physics package for use in miniaturized atomic clocks.

In terms of miniature atomic magnetometer, Zhang et al. proposed a compact 795 nm VCSEL-based atomic magnetometer based on a Rb microcell^159^. The configuration of the miniature atomic magnetometer applying VCSEL is shown in Figure 9c. The authors used a geometric-phase (GP)-lens to accomplish both the collimation of divergent beam and the selection of circularly polarized light. When the incident laser has an arbitrary polarization, the RCP σ+ component is focused into a collimated beam. This beam then passes through the vapor cell, serving as the pump light. On the other hand, the LCP σ- component is split and results in a defocused beam exiting the light path. Thus, this process purifies the circular polarization degree and effectively suppresses polarization noise. The GP-lens collimated VCSEL is applied in compact magnetometers. A square glass cell with external dimension 4 × 4 × 4 mm^3^ and interior dimension 3 × 3 × 3 mm^3^ is employed, containing a droplet of 87Rb. The photograph of VCSEL pump laser, GP lens, vapor cell, and the supporting structure are shown in the right of Figure 9c. The vapor cell is enclosed within a boron-nitride oven and subjected to a temperature of 150 °C. This is achieved by utilizing an electronic heater powered by a 200-kHz ac current. To ensure accurate temperature control, a nonmagnetic Pt1000 temperature sensor is securely affixed to the surface of the vapor cell. The temperature fluctuations are kept below 10 mK through the use of a real-time PID controller. Additionally, any magnetic field produced by the heating current can be counterbalanced by the tri-axis coils. High sensitivity up to 30 fT/Hz^1/2^ was achieved in this single-beam miniature atomic magnetometer.

According to the above-reported results, chip-scale atomic sensors offer a compelling combination of small size, high precision, stability, low power requirements, and scalability. These attributes make them attractive for various applications where accuracy, reliability, and integration are crucial, paving the way for advancements in sensing technology and enabling the development of innovative devices and systems.

### VCSELs with polarized beam emissions

In many applications, like atomic sensors mentioned above, high polarization stability is required for the light sources. However, the traditional methods of generating polarized beam often involve using bulky and expensive components like linear polarizers and quarter-waveplates. These methods also require meticulous assembly for proper functioning, posing a challenge to device miniaturization for portable and wearable applications. Hence, there is a pressing need for ultracompact polarized lasers that are simple to operate, enabling the development of highly integrated and cost-effective optical systems. VCSELs provide a promising platform to achieve this objective. However, conventional VCSELs generally have difficulty in achieving polarized light with a high orthogonal polarization suppression ratio (OPSR). The fundamental principle of polarization stabilization involves enhancing the desired polarization and suppressing other adverse effects to ensure its dominance. Adhering to this principle, various approaches, such as introducing anisotropic gain/loss^160,161^, and using asymmetry mesa^162^, have been explored to control the polarization of the output light.

**Fig. 10:**
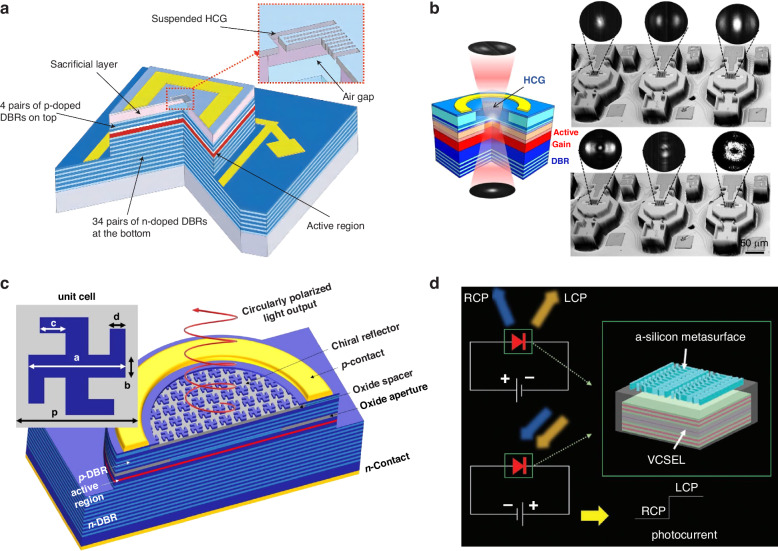
VCSELs with metastructures for polarization control. **a** Schematic cross-sectional layout of the VCSEL with the top mirror consisting of a freely suspending HCG and four pair DBRs^165^. **b** The cross section of a VCSEL with HCGs for beam shaping^166^. **c** Schematic of a VCSEL featuring a chiral metasurface as the top reflector for circularly polarized emission; the top-left inset shows a top view of the metasurface unit cell, with the geometric parameters labeled^167^. **d** VCSEL with a-Si metasurface on top facet. Device can simultaneously generate RCP and LCP beams when operating as a laser (forward biased)^168^. Device can also distinguish helicity of incident light when operated as a photodetector (reverse biased). Figure **a** is reprinted from ref. ^165^. with permission from the Springer Nature. Figure **b** and **c** are reprinted from ref. ^166^. and ref. ^167^. respectively with permission from the he Optica Publishing Group. Figure **d** is reprinted from ref. ^168^. with permission from the John Wiley and Sons

In addition to these methods, high-index-contrast metastructures offer a more flexible and effective way to achieve stable polarization while maintaining a high compactness^163,164^. High-contrast gratings (HCGs) are a type of optical metastructure used to manipulate light at the sub-wavelength scale. These gratings consist of alternating regions with high and low refractive indices, creating strong variations in the optical properties of the material. By carefully designing the periodicity and dimensions of the grating, HCGs can control the reflection, transmission, and diffraction of light. C. J. Chang-Hasnain group utilized HCGs with high reflectance and broad bandwidth to replace the DBRs of VCSELs to achieve stable polarization and solve the small DBR bandwidth problem (Fig. 10a)^165^. Since the HCG reflectivity is polarization-dependent, the device emission has a preferred TM-polarization with its electric field perpendicular to the grating lines, and an OSPR of 20 dB was achieved. In addition, this group tailored the angular transmission characteristics of HCGs to shape the angular profile of the VCSEL output beam, and realized single-lobe, double-lobe, triple-lobe, “bow-tie,” “sugar cone,” and “doughnut” beam patterns (Fig. 10b)^166^. Recently, the same group realized chiral lasing from electrically pumped VCSELs at room temperature without spin injection via incorporating a high-contrast chiral metasurface reflector as the VCSEL top mirror (Fig. 10c)^167^. When there is a difference in reflectivity between the top mirror for the two orthogonal circular polarizations, it leads to distinct threshold gain values for the two chiral modes. Consequently, the lasing behavior becomes dependent on circular polarization. More specifically, if the reflectivity of the top mirror is greater for RCP component compared to LCP component, the threshold gain for RCP component will be comparatively lower. As a result, the VCSEL tends to favor lasing in RCP polarization and vice versa. Based on this principle, the group designed the structure of the high-contrast chiral metasurface consisting of gammadion-shaped GaAs nanostructures arranged in a square lattice, sitting on top of a low-index aluminum oxide spacer. By adjusting the geometrical parameters (a, b, c, d) for the gammadion shape and the period of the square lattice (p) as shown in the inset of Fig. 10c, it is possible to control the circular-polarization-dependent reflection of the chiral metasurface. Through numerical simulations, it has been observed that the geometric parameter c, which corresponds to the length of the four orthogonally arranged arms of the gammadion shape, can be effectively utilized to manipulate the sign of the reflectivity difference. By setting proper geometrical parameters of (a, b, c, d), the VCSELs showed stable single-mode chiral lasing and achieved a circular-polarization degree of up to 59%.

Figure 10d shows a VCSELs with on-facet metasurfaces for polarization state generation and detection proposed by Wen et al.^168^ An amorphous silicon (a-Si) metasurface was fabricated and integrated on the VCSEL facet. When LCP and RCP waves interact with the amorphous silicon nanopillars, they undergo a separation process where each component is directed towards a different path due to the opposite phase gradients provided by the nanopillars. Additionally, the device can function as a detector by applying a reverse bias and illuminating it with light in the first-diffraction-order.

### VCSEL-based 3D photonic neural networks

With the rapid development of large models of artificial intelligence (AI), the problems of insufficient computing power and excessive energy consumption of existing von Neumann architecture computers have emerged^169,170^. Taking ChatGPT-3 for example, it contains 175 billion parameters, and the total computing power needed for training is 3.14 × 10^23^ flops^171^, which requires running on 1,024 NVIDIA A100 graphics processing units (GPUs) for up to one month. In this context, neuromorphic computing mimicking the nervous system of the human brain has become a widely recognized solution^172^. Among them, photonic neural networks (PNNs) have significant advantages over their electrical counterparts in terms of power consumption and speed because of their light-speed passive propagation, high bandwidth, low crosstalk, and multiple physical dimensions^173–176^. Bhavin J. Shastri et al. estimate that the energy efficiency and computing power of the PNNs based on nanophotonics will be 4 orders of magnitude and 5 orders of magnitude higher than the state-of-the-art neuromorphic electronics, respectively^177^.

Spatially, PNNs can be categorized into two- and three-dimensional (2D and 3D). 2D PNNs mainly refer to photonic integrated circuits represented by Si-photonics technology. So far, integrated 2D PNNs including deep neural networks^177,178^, spiking neural networks^179,180^, tensor cores^181^, and convolutional accelerators^182,183^ have been demonstrated. However, due to the working wavelength, the synaptic device size of 2D PNNs typically reaches the micrometer level, resulting in a much lower device density than current electronic integrated circuits. Because of the limited chip size, 2D PNNs cannot achieve large-scale device integration. Thus, despite better energy efficiency, the computing power of 2D PNNs is difficult to surpass existing commercial GPUs.

3D PNNs are a promising way forward. By increasing the spatial dimension, the number of devices can be greatly increased to improve the performance. In electronic circuits, increasing the spatial dimension involves complex circuit design and fabrication, whereas in PNNs, this problem is drastically simplified. Light can propagate on demand in free space using conventional DOE devices^184–186^, optical fibers^187–189^, or holography^190–193^. VCSELs have played an important role in 3D PNNs. The unique characteristics of VCSEL, including 2D array, addressability, low threshold, and high modulation bandwidth, enable it to meet the high demands of 3D PNNs for high-throughput input, low energy consumption, and compactness.

**Fig. 11:**
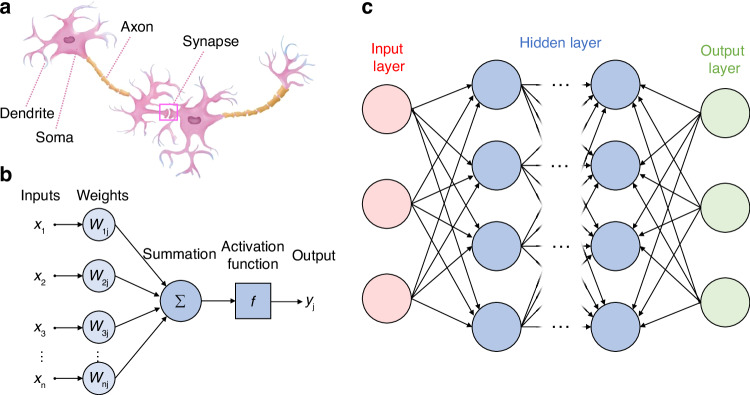
Typical artificial deep neural network principle. **a** Schematic illustration of a biological neuron^170^. **b** Schematic illustration of the mathematical model of a biological neuron. **c** Schematic illustration of a typical framework of a fully-connected deep neural network. Figure **a** is adapted from ref. ^170^. with permission from the Springer Nature by CC BY 4.0

Figure 11a, b show a schematic drawing of a biological neuron and its mathematical model, respectively. The weight function of synapses, the summation function of soma, and the nonlinear functions are key elements in building PNNs. Mathematically, the weight and summation functions can be represented by matrix multiplication. Figure 11c is a typical framework of a fully connected deep neural network with an input layer, multiple hidden layers, and an output layer. Each hidden layer consists of many neurons carrying different weights. When the input signal in matrix form passes through a hidden layer, it performs matrix multiplication with the weight matrix stored in the hidden layer. Matrix multiplication includes multiplication operations and summation operations, which can simulate the functions of synapse and soma respectively.

The advantages of VCSELs to be used in PNNs and/or optical computing can be broadly categorized as follows. (1) As active devices, VCSELs can be pumped and lasing by optical injection^194,195^. The pumped laser can be modulated electrically^196^ or by introducing other materials, like saturable absorber^189,197^. Therefore, VCSEL can accomplish the weight function or the summation function and have been used as photonic neurons in deep neural networks, spiking neural networks, and reservoir computing. (2) The optical pumping of VCSEL reacts highly nonlinearly to optical injection^198^, so VCSEL-based PNNs naturally have a nonlinear activation function. (3) Electrically driven VCSELs also can be used as input layers to generate high-throughput signals for PNNs^199^. (4) In superconducting processors and quantum computing, low-temperature environments are often required. VCSELs can be designed to operate stably at low temperatures, and the high-speed feature of VCSELs allows them to be used for data transfer from ultralow temperature to the end-user at room temperature^200^.

#### VCSEL-based deep neural networks

**Fig. 12:**
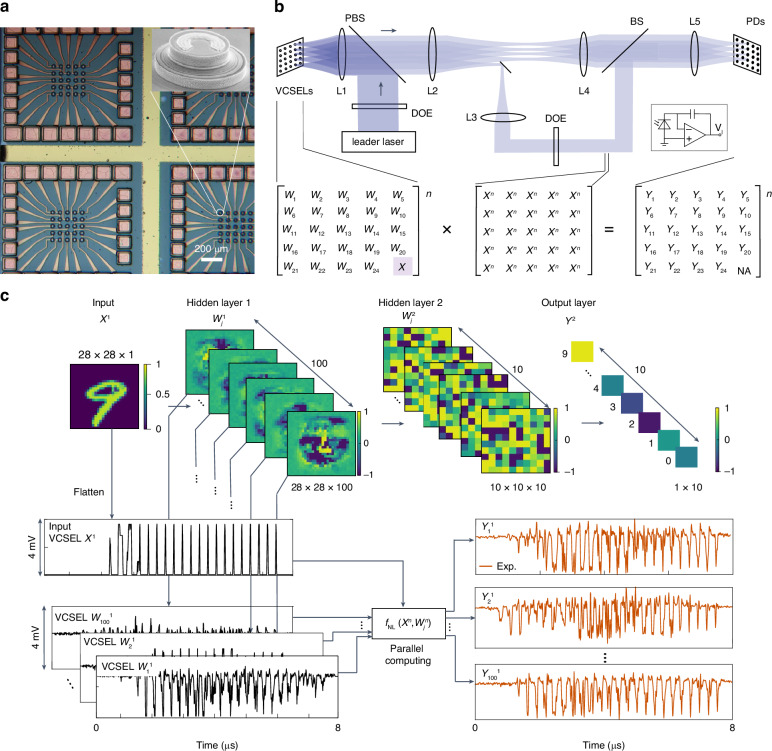
Deep PNNs based on coherent VCSEL array^184^. **a** Optical image of the used VCSEL array. The inset: scanning electron microscope image of a VCSEL. **b** Schematic illustration of the optical setup. **c** Benchmarking of optical inference with the VCSEL-based deep PNNs. Figure **a** is adapted from ref. ^184^ with permission from the Springer Nature. Figure **b** and **c** are reprinted from ref. ^184^ with permission from the Springer Nature

Recently, Chen et al. reported an optical deep neural network based on a 5×5 coherent VCSEL arrays (Fig. 12a)^184^. The synaptic weight function is realized by controlling the phase difference between the VCSELs. First, they achieved coherence of VCSELs by optical injection phase-locking. As illustrated in Fig. 12b, a leader laser was illuminated on the VCSELs by a DOE device. Therefore, the VCSELs serve as the slave lasers. When the frequency detuning between VCSELs and the leader laser is in a certain range, the phase of all the VCSELs will be locked to the leader laser. Thus, the VCSELs become coherent. The locking range, i.e. the range of frequency detuning, is related to the injection ratio, which is defined as the ratio of output power of the VCSELs and power of the injected signal^195^. As the ratio increases, the locking range increases. Then, under injection locking, tuning the individual VCSEL resonance with varying driving voltages allows phase tuning. In their work, the voltage range V_π_ to achieve a phase shift of (−π/2, π/2) is 4 mV, and such a small V_π_ allows phase-only linear modulation with negligible amplitude coupling. Therefore, all the data, including input signals and weights can be encoded as the phase of VCSELs.

During the computing, a single VCSEL in the array served as the signal input and was encoded by sequential input signals *x*. Then, the signals were fanned out to multiple light spots by another DOE device and illuminated on multiple photodetectors respectively. Other VCSELs served as the weights encoding different sequential weights *w* and were also illuminated on these photodetectors respectively. As a result, the photocurrent *I* of one of the photodetectors satisfies the following relationship,

1 $$I\propto {A}_{w}{A}_{x}\sin ({\varphi }_{w}-{\varphi }_{x})$$

where A_x_, A_w_, *φ*_*x*_, and *φ*_*w*_ are the amplitude and phase of the input and weight VCSEL, respectively. Since the information is encoded in the phase, which means the amplitude of the VCSELs can be regarded as a constant, this equation can be rewritten as

2 $$I\propto \sin \left({\varphi }_{w}-{\varphi }_{x}\right)=w\sqrt{1-{x}^{2}}-x\sqrt{1-{w}^{2}}$$

where $w=\sin {\varphi }_{w}$ and $x=\sin {\varphi }_{x}$. Thus, the synaptic weight function is successfully realized. The circuit of the photodetector was designed to accumulate the photocurrent generated by the sequential optical signal, thus realizing the summation function of soma.

Based on the above mechanism, the authors trained a bilayer deep neural network and implemented it through two rounds of calculations on the VCSEL array (Fig. 12c). The authors demonstrated recognition tasks of different datasets. Among them, the accuracy of 10-type handwritten digit recognition can reach 93.1%. With optimized electronics and photonic packaging, the computing power and energy consumption of the optical part of the network can reach 6 TeraOP/(mm^2^·s) and 7 fJ/OP, respectively, which are improvements of 100× and 20× compared to digital hardware.

#### VCSEL-based spiking neural networks

In the human brain, biological neurons are excitable and respond by firing spikes when stimulated^201^. The spiking neural networks (SNNs) mimic this model and are known as the third-generation artificial neural network. In SNNs, signals are transmitted in the form of spikes rather than simulated continuous values^179,180^. The leaky integrate and fire (LIF) model is one of the most commonly used models for spiking neurons. In the LIF model, the spiking neuron integrates the stimulus. When the accumulation exceeds the threshold, the spiking neuron will be activated and generate a spike to pass backward^188^.

VCSEL can be used as an optical LIF spiking neuron. In 2012, A. Hurtado et al. reported the spiking behavior of VCSELs under optical injection and correlated VCSELs with spiking neurons^202^. Compared to biological neurons, the optical spiking phenomenon of VCSELs is several orders of magnitude faster, typically at the picosecond level^202^, which helps to significantly increase the processing speed. Parallelly, excitable spiking characteristics of VCSELs containing intracavity saturable absorbers have also been investigated^197^. This phenomenon can be simply understood as an increase in carrier concentration in the active layer of the VCSEL caused by optical injection, which is similar to the summation function of soma. When the carrier concentration exceeds a certain threshold, the VCSEL can be pumped, and due to the recombination of holes and electrons, the carrier concentration drops rapidly and falls below the threshold. As a result, spike-shaped laser pulses are generated by the VCSEL.

**Fig. 13:**
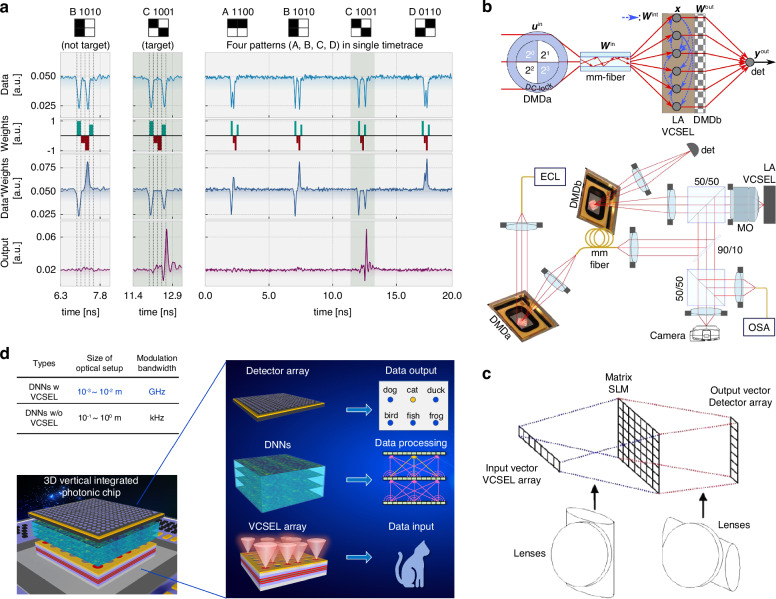
Application of VCSEL in different PNNs. **a** 2-bit image recognition based on spiking VCSEL-neurons^188^. **b** Reservoir computing based on LA-VCSEL^198^. **c** Matrix multiplication using VCSEL as 1D vector input device^236^. **d** 2D VCSEL array can be used as high-through-put input device for DNNs to implement 3D photonic chips^199^. Figure **a** is reprinted from ref. ^188^. with permission from the Springer Nature by CC BY 4.0. Figure **b** is reproduced from ref. ^198^. with permission from the IOP Publishing by CC BY 4.0. Figure **c** is reprinted from ref. ^236^. with permission from the Optica Publishing Group. Figure **d** is reprinted from ref. ^199^. with permission from the De Gruyter by CC BY 4.0

Recently, spiking VCSEL-neurons have been used for different tasks, such as logic operations^203^, image edge detection^204^, convolution kernel^205,206^, and pattern classification^188^. Here we select the pattern classification task to introduce. In the work^188^, as shown in Fig. 13a, a 4-bit pattern is encoded as time-sequential optical signals. After being weighted by a Mach-Zehnder modulator, only the sequence of the target pattern can produce two stimulus pulses within the integration time of the VCSEL. The used VCSEL could only be activated by two pulses stimulated during the integration time. Therefore, when these optical signals are injected into the VCSEL, only the signals of the target pattern can activate the VCSEL to produce a spiking response. Based on the high-speed characteristic of VCSEL, the recognition of a 4-bit pattern only takes 650 ps.

Currently, VCSEL, as a spiking neuron, mainly plays the role of integrating optical signals. Optical signals are mainly transmitted through optical fibers, therefore, the number of VCSEL neurons used is limited, resulting in the demonstrated tasks being generally simple. In the future, if the network connections of spiking VCSEL-neurons can be constructed through DOE devices, it may be possible to construct large-scale SNNs to implement more complex AI tasks.

#### VCSEL-based reservoir computing

VCSEL can also be used to build recurrent reservoirs to implement reservoir computing^199,207–209^. The reservoir is a fixed, nonlinear system with an internal dynamic process that maps the input signal to a higher-dimensional computational space. The reservoir can be regarded as a “black box”. We do not need to care about the signal transmission and connection in it and only need to train the readout weights to process the output. In the work shown in Fig. 13b, the authors used a large-area VCSEL (LA-VCSEL) as reservoir^198^. The LA-VCSELs indicating the VCSELs with large emitting surface, are multimode. This type of VCSEL can implement all the components of the reservoir through their spatio-temporal nonlinear dynamics. Here, the input optical signals are generated by a digital micromirror device (DMD) and transmitted through a multi-mode fiber. During this process, the signal is implemented with complex input weights because of the optical mode mixing induced by birefringence and scattering. Then, the signals are injected via imaging onto the LA-VCSEL top-facet. The optical signals illuminate at different locations, representing the nodes of the reservoir, of the emitting surface of the LA-VCSEL. Optical diffraction, as well as carrier diffusion in the LA-VCSEL intracavity field, induces interactions between different locations, i.e., coupling between the photonic nodes. Thus, a reservoir is formed inside the LA-VCSEL. Afterward, the pumped optical signals from the LA-VCSEL are then transmitted to another DMD encoded with output weights. Finally, the outputs are captured by a large-area detector. Based on this architecture, they demonstrated the tasks of 2-bit digit recognition and XOR.

In addition to the above work, VCSELs have been widely used to construct spiking reservoir computing^210^. Besides, micropillar VCSELs can also be for reservoir computing^75,211^. For more information about VCSEL-based reservoir computing, please refer to these reviews^187,210^.

#### VCSEL-based optical input layer

Through the design of circuits, an addressable VCSEL array can become an ideal laser source serving as an input layer for generating optical data. VCSEL can be used to implement matrix-vector multiplication (MVM). MVM occupies the most of computing power in AI tasks. In models, such as GoogleNet and OverFeat, more than 80% of computing is MVM^212^. Therefore, realizing MVM through optical methods is of great significance. As early as 1978, J. W. Goodman et al. had demonstrated optical MVM through light-emitting diodes^213^. After the 1980s, VCSEL began to be used in MVM^214–217^. In VCSEL-based MVM, 1D or 2D VCSEL arrays are used to generate optical input signals. A basic optical setup is shown in Fig. 13c. The generated signals by a one-dimensional (1D) VCSEL array are first fanned out by a DOE device and multiplied with the transmittance matrix and then fanned in by another DOE to implement addition operation. Finally, these signals are captured by a detector array, thus realizing the multiplication of a 1D vector and 2D matrix.

In addition to being used in MVM, VCSEL can also be used in diffractive neural networks (DNNs)^199^. First reported in 2018, DNNs based on optical holography consist of cascade diffractive layers^190^. Each pixel in the layer can be regarded as a neuron. Based on optical diffraction, neuron connections can be constructed without DOE devices. In this work^190^, the authors successfully demonstrated handwritten digit recognition with a 5-layer DNN, and the accuracy can reach 88%. Subsequently, based on different implementation technologies of the diffractive layers, reconfigurable DNNs^193^, programmable DNNs^218^, and integrated DNNs^219–221^ were successively realized. DNNs can directly process optical images, thereby enabling high throughput data processing. Currently, DNNs have been widely used in a variety of AI tasks, such as recognition^222,223^, beam shaping^224^, lensless imaging^225^, and phase imaging^226^.

However, one of the bottlenecks of DNNs is the slow speed of the signal-generation devices. At present, the optical image signals processed by DNNs are mainly generated by a SLM, DMD, or masks, which have a low modulation rate, thereby greatly limiting the real computing power of the DNNs. The high modulation speed of VCSEL just meets this high demand. Recently, Min Gu et al. published a perspective article detailing the huge role that VCSELs may play in DNNs^199^. They proposed a VCSEL-based 3D photonic chip architecture shown in Fig. 13d and discussed the potential developments, including spiking DNNs, multiplexing DNNs, and programmable DNNs. As well as they put new requirements for VCSELs to be used in DNNs.

## Conclusions and outlook

VCSELs possess unique advantages in the field of integrated photonics, including perpendicular emission, low power consumption, high temperature stability, high direct modulation speed, scalable two-dimensional arrays, and ease of monolithic integration. By utilizing VCSEL as a laser source and integrated platform, it is possible to directly integrate micro/nano functional optical components such as metasurfaces, phase plates et al., enabling various chip-level versatile photonic devices and systems. This integration allows the transfer of the charming inherent characteristics of VCSELs to the integrated devices and systems, creating great potential for the next generation of highly integrated, portable, high-performance, and low-power optoelectronic systems. In the context of VCSEL-based integrated devices, metasurfaces demonstrate powerful wavefront manipulation capabilities, which can enable multi-functionalities including beam control, polarization control, holographic imaging and generation of vortex beams, and so on. Furthermore, the ultrathin two-dimensional structure of metasurfaces makes them unparalleled in the field of miniaturized integrated photonics. Future efforts need to address the challenges of high-yield and large-scale manufacturing of metasurfaces.

In this review, we summarized and discussed recent developments in VCSEL-based integrated photonic devices and systems, including photonic neural networks, vortex beam emitters, holographic devices, beam deflectors, atomic sensors, and biosensors. These advanced integrated devices based on VCSELs exhibit strong appeal in specific domains. For instance, VCSEL-based photonic neural networks offer powerful parallel computing capabilities, ultra-low power consumption, and extremely fast operation speeds, making them promising alternatives for post-Moore computing architectures. Another example is VCSEL-based vortex beam emitters, which can easily achieve multi-channel vortex beam arrays due to the direct high-speed modulation capability of VCSELs and their addressable and scalable two-dimensional arrays. By utilizing vortex beam multiplexing techniques, these devices can significantly enhance optical communication capacity, which will become highly attractive in future large-capacity optical communication applications once the long-distance transmission challenges of vortex beams in optical fibers are solved.

To better support the development of VCSEL-based photonics, VCSELs may need to be further developed in the following aspects. First, there is a need to further develop long-wavelength VCSELs covering from 1.3 to 2.3 μm in a more cost-effective and mass-production way, which is very important for applications such as silicon PICs, long-reach data communications, and gas sensing. In the short-wavelength regions, ultraviolet (UV) VCSELs operating below the 400 nm wavelength are attractive for applications ranging from disinfection to compact ytterbium-ion based atomic clocks, and have great potential to be used in the state-of-the-art 3D laser nanoprinting^227^. Efforts should be made in developing electrically pumped UV VCSELs. Second, developing larger-scale addressable coherent VCSEL arrays with high modulation bandwidth is necessary for PNNs. To solve the problem of addressing circuits, it is necessary to consider using back-emitting VCSELs flip-chip bonded on the integrated circuits. In addition, in large-scale VCSEL driving, to ensure the high stability of VCSEL, heat dissipation issues need to be considered. Third, new types of surface-emitting lasers with improved performance should be developed and considered as light sources for integrated photonic devices and systems to overcome certain limitations of VCSELs and extend their functionalities. For instance, photonic-crystal surface-emitting lasers (PCSELs) offer advantages such as higher single-mode output power exceeding 10 W, extended wavelength range, and much smaller divergence below 0.5 degree^228–230^. In addition to PCSEL, micro/nano surface-emitting laser arrays with high-performance are also attractive laser sources for communication, computing and spectroscopy applications^231–235^. By leveraging and extending the capabilities of VCSELs, these integrated photonic devices/systems will open up new opportunities in various fields, including artificial intelligence, large-capacity optical communication, imaging, biosensing, and so on.
